# Women’s experiences and needs concerning care and support during the various phases of childbirth and the postnatal period: Analysis of free-text comments based on Quality from the Patient’s Perspective in Sweden

**DOI:** 10.18332/ejm/176698

**Published:** 2024-02-19

**Authors:** Karin Ängeby, Elin Ternström

**Affiliations:** 1 Women's Department and Centre for Clinical Research Education, County Council of Värmland, Karlstad, Sweden; 2 School of Health and Welfare, Dalarna University, Falun, Sweden; 3 Department of Women’s and Children’s Health, Uppsala University, Akademiska Sjukhuset, Uppsala, Sweden

**Keywords:** labor phases, women-centered care, quality of care from patient’s perspective, free-text comments

## Abstract

**INTRODUCTION:**

Positive birth experiences can be a decisive factor in the well-being and future health of both women and their newborns. The quality of care is a multidimensional concept influenced by the external structure of the organization, the administrative qualities of the environment, and the individual patient’s preferences about care. The aim was to describe women’s preferences and experiences concerning support and treatment, and their perception of quality of care during all phases of labor and the postnatal period.

**METHODS:**

Free-text comments of 635 women from four different open comment questions were analyzed. A qualitative content analysis was conducted in two steps: an inductive phase followed by a deductive phase using the Quality of care from a Patient’s Perspective framework (QPP).

**RESULTS:**

A total of 1148 free-text comments were coded; and 10 sub-categories were created and inserted under the QPP framework covering the latent meaning of the sub-category. Five of the sub-categories were sorted under the identity-oriented approach, four under physical-technical conditions, and one under the sociocultural atmosphere and reflected the women’s experiences and needs regarding support and treatment during early labor, the active phase of labor, and the postnatal period.

**CONCLUSIONS:**

High-quality care and support are important aspects for women during childbirth, irrespective of the phase of labor or postnatal period. The need for individualized care, active participation in one’s own birth and using a family centered approach were also emphasized. Organizational factors influenced the quality of care and were particularly noticeable during birth.

## INTRODUCTION

Giving birth, a central event in a woman’s life is both psychologically and physiologically challenging^1^. A woman’s birth experience can be a decisive factor in her future well-being. During the different phases of childbirth, the need for support can vary, and each woman has different needs for care. The WHO (2015) states that the quality of care for women should be safe, effective, timely, efficient, and women-centered^2^ and is recognized as a critical aspect of both maternal and newborn health^3^. The quality of care is a multi-dimensional concept influenced by the organization’s external structure, the environment’s administrative qualities, and the individual patient’s preferences. Birthing women state that their health professionals shall combine their clinical knowledge and skills with interpersonal and cultural competence^4^.

Labor and birth are traditionally divided into phases and stages^5-7^. For women, it can be an emotional journey towards birth instead of fixed phases and stages^8^, of which the postnatal phase is an integrated part of the laboring experience^9^. As maternity care has developed, support and presence from professionals have also changed^6^. The postnatal period is an underserved aspect of maternity care, and postnatal care guidelines are not tailored to meet the essential needs of women. A meta-synthesis has revealed that women also want to feel ‘cared for’ during the postnatal period, emphasizing good quality care and flexibility regarding care^10^. The length of postnatal stay and the type of care are related to satisfaction, according to the findings of a Swedish study. The most critical variables for being ‘very satisfied’ with postnatal care were high-quality medical care for the infants and sufficient support from staff for the mother^11^.

The theoretical model of quality of care from the patient’s perspective (QPP) was based on a grounded theory study^12^ and adapted to intrapartum care QPP-I^13^. The resource structure of the care organization is two-fold: first, the person-related qualities of the caregivers, and second, the physical and administrative environmental qualities that refer to the infrastructural components of the care environment, such as organizational rules and technical equipment. Therefore, the patient’s perceptions of the quality of care may be examined on four dimensions: the medical–technical competence of the caregivers, the physical–technical conditions of the care organization, the identity–oriented approach of the caregivers, and the sociocultural atmosphere of the care organization^12^. In Sweden, maternity care is publicly funded and free of charge. The majority of women choose to give birth in hospitals. Midwives provide care for all women in the labor ward and act independently as care providers in uncomplicated labor and birth^14^.

Midwives’ support and care are important for promoting positive birth experiences for women. When the midwife has limited opportunities to create a positive relationship with the woman or the support is lacking, the birth experience can be affected. To deepen and enhance knowledge about women’s experiences of support and care they received during their childbirths, we conducted an analysis consisting of free-text comments from a questionnaire exploring women’s experiences of labor care quality. This study aimed to describe women’s preferences and experiences concerning the support and treatment received and their perceptions of the quality of care during all phases of labor and the postnatal period.

## METHODS

### Design and setting

The analysis was performed using qualitative content analysis with an inductive approach, described as advantageous for text that requires analysis, processing, and interpretation^15^, followed by a deductive approach^16^ based on the dimensions of QPP-framework^12^. The article adheres to the Standards for Reporting Qualitative Research (SRQR) checklist for reporting qualitative studies^17^ (Supplementary file).

### Participants

A one-year cohort of women who had given birth between 1 September 2013 and 31 August 2014, was invited to take part in the study. It was carried out in Central hospital, in the rural county of Värmland, located in the western part of Sweden. The labor ward surveyed was of average size, the only one in the county, and had approximately 2700 births per year. Primiparous and multiparous women with spontaneous onset of labor were invited to respond to the questionnaire as described above. The guidelines of the labor ward at the hospital encourage women to return home if they are not in the active phase of labor. Instead of going home, the women can stay at a closely situated patient hotel if they prefer, but without midwife support. The postnatal care takes place at the postnatal ward, at the patient’s hotel, or at home. In total, 771 women answered two questionnaires, QPP-I and the Early Labor Experience Questionnaire (ELEQ) and additional questions related to childbirth, with a response rate of 63%. The respondents included 353 primiparous and 418 multiparous women. The majority had a vaginal birth (90.2%), had a mean age of 31.3 years, and 55% had a university degree.

### Data collection and analysis

The study was conducted using a qualitative design based on free-text comments. The data originated from a major research project in which women’s experiences of early labor and quality of care were examined^18,19^. The questionnaires used in the present study were Intrapartal Quality from the Patient’s Perspective (QPP-I)^13^ and the ELEQ. The questionnaire had a total of 57 questions. The majority were multiple-choice questions, but there was also a possibility of leaving free-text comments. The possibility to write free-text comments was located in four different sections in the original questionnaire. The first space for the free-text comments was placed after questions about healthcare contacts in early labor and after the question ‘Were you satisfied with leaving the labor ward in early labor?’, and marked as a place for comments. This space received responses from 87 women. The second space was labelled ‘This was what I was delighted with’ and was placed after the QPP-I questions (as in the original QPP-I questionnaire), which 575 women responded to. The third space, labelled ‘Suggestions of improvements’, was placed in connection with the second space and received responses from 392 women. The fourth space for free-text comments, marked as just ‘Comments’, was inserted after ELEQ questions and responded to by 253 women. In total, 1307 free-text comments were checked, and 1148 free-text comments concerning women’s experiences and needs regarding care and support were consolidated for further analysis. Qualitative content analysis using an inductive approach was used to analyze the open comments. In Step 1, patterns in the data were identified without a predetermined theoretical framework^15^. All the authors read the comments several times to gain a general overview of the responses. Sentences or phrases corresponding to the aim were selected and divided into units of meaning. The meaning units were processed into condensed units. The condensed meaning units were abstracted into codes that described the meaning units using shorter and more descriptive formulations. Using color coding, the codes were grouped based on content. The color codes were sorted into categories. The created categories reflected the central message of the findings^15^.

In Step 2, to emphasize the latent meaning of the categories, the authors deductively sorted the categories created in the inductive phase^16,20^ into four different dimensions of the framework of quality of care^21^ to establish the framework of quality of care from the patient’s perspective.

### Ethics

This study was approved by the Regional Ethical Review Board in Uppsala, Sweden (No.2012/490). All the participants were informed that participation was voluntary and gave their informed consent. They were also informed of their right to withdraw at any time without affecting their care.

## RESULTS

The findings were based on free-text comments that reflected the women’s experiences and needs regarding support and treatment during early labor, the active phase of labor, and the postnatal period. In total, 635 women responded to any of the free-text spaces. The women who responded to the free-text comments were significantly more often primiparous women (87.0% vs 77.5%; p<0.001) and more often had an instrumental birth (10.9% vs 2.9%; p=0.002). However, no significant differences were found regarding positive birth experience, country of birth, or education level.

All ten sub-categories were covered under the domains of the QPP framework (Table 1). Five sub-categories were sorted under the identity-oriented approach, four under physical-technical conditions, and one under a sociocultural atmosphere. No sub-categories were sorted in the medical-technical competence domain.

**Table 1:** Women’s experiences and needs concerning care and support during the various phases of childbirth and postnatal period: categories sorted under the quality of care from the patient’s perspective framework

*Identity-oriented approach*	*Medical-technical competence*	*Physical-technical conditions*	*Sociocultural atmosphere*
The importance of being able to influence the timing of hospital stay	-	Insisting on high quality care both when staying at the hospital and the patient hotel	Strong emphasis on family-centered care
Support and presence during all the stages of labor and birth	-	Having environmental wishes fulfilled	
Needing individualized and nuanced information	-	Being affected by stressful work situations	
To be taken seriously and involved as an active participant in one’s own childbirth	-	Desiring the continuity of carers and a functioning team	
Empathetic support and care	-		

### The importance of being able to influence the timing of hospital stay

Being able to influence both when to arrive at the labor ward and when to leave the postnatal ward was necessary for the women. They did not wish to decide when to arrive or leave by themselves but desired to make informed choices and feel involved and supported in their decisions. The first contact was often established by telephone, and the autonomy in decision-making, with a midwife’s support regarding whether to stay at home or go to the hospital, was perceived as positive by some women:

*‘The midwives were professional and nice! They focused a lot on the fact that it was my experience that should decide when it was time to go in, an excellent perspective.’*

For other women, however, this choice caused anxiety and an experience of being left out with the responsibility of tough decisions to make. The women who visited the labor ward for check-ups and actively participated in the decision to stay or go home, reported increased satisfaction and feeling included in the decision-making process. Having to leave the hospital without their consent could create immense anxiety and insecurity. Several women reported that the long distance between their homes and the hospital reinforced their feelings of insecurity. Some women felt that since they needed to leave the hospital, they did not get adequate pain relief in the labor ward. The need to be active in deciding the length of hospital stay was also notable during the postnatal period when women expressed the desire to be more involved in decisions about when to leave the hospital. Some women described experiences in which the staff were in a hurry to send them home from the hospital, which resulted in women going home without the support and care they had wanted and needed.

### Insisting on high quality care both when staying at the hospital and at the patient’s hotel

Regardless of where the women were admitted when seeking care in early labor or requiring care postnatally, high quality midwifery care was strongly preferred, which consequently instilled a sense of security. That experience could be decisive for their overall birth experience:

*‘I went to the hospital with enormous pain, but my cervix hadn’t dilated anything. Was still nicely treated and could stay in the ward as there was room for me. Very happy with this and think that was quite crucial for my positive experience.’*

Some women who stayed at the patient hotel close to the labor ward described the patient hotel as a safe alternative. Others expressed that the sense of security at the patient hotel would have been even greater with an onsite midwife. During the postnatal period, the women also expressed the desire for increased security at the patient’s hotel:

*‘Midwives at the patient hotel!! I felt very abandoned and left alone at the patient’s hotel. I wanted to have more support during breastfeeding!’*

### Support and presence during all stages of labor and birth

Women expressed that the presence and support of midwives were essential during all stages of labor and the postnatal period. They wanted to be treated as individuals with individual needs and to feel prioritized by midwives, even though the midwives often had other tasks and patients to care for.

During early labor, most women expressed wanting more support from midwives. Their sense of security increased when the midwife was present and provided individualized support. The women’s comments also reflected the importance of the midwives’ support and presence during early labor pain at the hospital. During the active phase of labor, many women emphasized how the presence of a midwife meant that they felt supported and involved in their care. They wanted continuous support and wished the staff were always there, not only when the woman or her partner called for them:

*‘The staff’s treatment and support and that they “steered” the situation in such a fantastic way when you felt that you lost your grip. Wonderful and understanding people who made me feel I was always in focus.’*

The midwives’ support and presence also remained important after birth. The women expressed that the midwife, the primary support system, disappeared after the child was born. The absence of staff created insecurity, and several women stated that they wanted increased supervision by the staff after birth. Above all, most of the women wanted more support for breastfeeding, significantly to help with latching. Notably, some women felt positive about the staff who did not comment or provide breastfeeding advice.

### Needing individualized and nuanced information

The women described the importance of straightforward and clear information during all phases of labor. When the woman had difficulty understanding and interpreting what was happening to her body, unclear information was troublesome. The lack of uniform information also led to decreased trust. Clear and well-adapted information was crucial to the relationship between the women and midwives. Women specifically expressed the need for continuous information on their progress in the birthing process, if the baby’s heartbeat was as expected, and the possible methods for pain management. The importance of obtaining information before and detailing various interventions has also been emphasized. The fact that nursing and medical interventions were performed without providing necessary information caused both pain and insecurity. Several women expressed that they did not feel adequately informed or that the information they received was individualized to their needs:

*‘More information. I often felt that everything was obvious and implied among the staff, but it was unclear how everything should work out for me as a first-time mother.’*

During the postnatal period, the women needed more information and discussions about childbirth experiences. It was crucial to talk in peace and quiet about the birth with the attending midwife and get their questions answered. Some women described follow-up phone calls after birth as immensely helpful in processing the labor experience:

*‘That the midwife who assisted me at birth took the time to talk to us immediately after the birth and at the postnatal ward was important. These conversations have been crucial for me in experiencing my childbirth as an okay experience instead of a trauma.’*

The women also requested more information on breastfeeding, caring for newborn babies, perineal tears, and the stitches. Several women mentioned the importance of consistent information and described how confusing and sometimes disturbing it was to receive inconsistent information from different midwives.

### To be taken seriously and involved as an active participant in one’s childbirth

Being taken seriously was very important for the women during their interactions with staff at the labor ward. At first contact, usually by telephone, several women described how the midwife’s communication often felt vague, incomprehensible, and incongruent with the women’s experience of the progress of the birth, resulting in feeling unheard by the staff. Women who gave birth to their first child felt that the midwife’s preconceived notions about their birth process resulted in their problems not being taken seriously. Instead, if the women were trusted, they described relief and satisfaction when their wishes were taken seriously and listened to.

During the active phase of labor, several women described how they desired increased sensitivity from the staff at the labor ward. Most women described being met with indifference in their perceived experiences during childbirth, and some women described how their decreased confidence created a feeling of diminution:

*‘I would have liked the staff to trust my feeling of the speed of the birth in the end. It felt like my feelings were overlooked and that they instead trusted their routines and how a birth should be.’*

When women were allowed to participate in the decisions made during birth, increased satisfaction and feelings of being taken seriously emerged. Some women also stated that they appreciated the staff stepping outside their comfort zones to satisfy the women’s wishes:

*‘That the midwife had a lot of eye contact with me and tried to read me often, that she listened to my wishes and respected them, that she was calm and thoughtful like me.’*

For women who had written birth plans that the staff participated in and respected, the positive birth experience was strengthened. These staff members were described as confident, responsive, cooperative, and supportive leaders. However, when the staff did not follow the birth plan, feelings of insecurity were possible.

### Empathetic support and care

When the first telephone contact was welcoming and encouraging the woman to come to the labor ward according to her wishes, the birth experience was more positive. However, several women described the inadequate treatment during the telephone conversations, with a general tone of ignorance, lack of empathy, and lack of individual counselling, highlighting their overall experiences:

*‘The midwife I first spoke to on the phone was very brusque and unpleasant, felt no confidence in her, and I did not become much wiser after asking my questions.’*

When women experience unpleasant treatment during early labor, feelings of rejection could arise. Some women described how the staff wanted to send them home, even before any vaginal examination, resulting in feeling unwelcome.

During the active phase of labor, most women were satisfied with the support and care they received. Feeling safe was important for positive birth experiences. It was promoted by personalized approaches, where the woman was seen as a unique individual and treated with expertise, calmness, and empathy, thereby becoming the building blocks for a good relationship:

*‘The staff was fantastic! My childbirth is something I only associate with something very positive. The staff’s treatment and competence contributed to us feeling safe and gave me so much strength during the birth.’*

When the labor staff included the partner during birth, the women described the treatment as positive. Acts of closeness, support, and massage have also promoted good treatment. However, some women described shortcomings in how they were treated by staff, who were uninvolved and impersonal. Some women described how the midwives’ touch during examinations was harsh. Most women who had bad experiences with the staff described the treatment as bad at night, where the staff members were arrogant, and some women described their experiences of being in the way.

During the postnatal period, several women described the staff as competent, and postnatal care was characterized by professionalism, warmth, and a caring atmosphere. However, for some first-time mothers, the need for more help with breastfeeding was evident. They also wished for more staff availability. The staff could also be perceived as careless and cold, and some women described feeling burdensome to the staff. When the staff felt uncertain about the routine in the clinic, some women felt insecure.

### Having environmental wishes fulfilled

Women described the need for certain attributes and equipment at the labor and postnatal wards but also wanted more alternatives for the whole birth environment. The desired alternatives to technocratic regular birth rooms were rooms that focused more on physiological births rather than potential risks and complications:

*‘I really wish there was a not-so-medical alternative to giving birth, while still being in the hospital, a little cozier and more natural.’*

Most women described that privacy during and after birth felt important and how the lack of their own shower and toilet at the labor ward could create feelings of vulnerability. Other wishes were better access to good food for both the woman and her partner, and aids that were helpful both during and after the birth, such as a Pilate ball, birthing stool, movable nitrous oxide, plastic pram for the new-born baby, television sets, and sanitary napkins, underwear, and changing tables for each room.

### Being affected by stressful work situation

At first contact with the labor ward, several women described the staff as stressed with high workloads. A high workload could create insecurity among the women who visited the ward, and some women described being fearful of not getting the help and support they needed during the birth:

*‘Our first contact at the labor ward was with an assistant nurse who we experienced as stressed and who expressed that they were understaffed. This made me feel insecure and I was afraid that we would not get enough help and support.’*

Some women described sufficient staffing but an atmosphere characterized by high workloads, which in turn negatively affected the birth experience. Some women also described their experiences of being in the way and feeling unwelcome. During the active phase of labor, several women described the stressful work environment as contributing to a loss of both support and presence in the labor room. Some women described being left alone often, making them feel insecure. The high workload was described as noticeable and also reflected in delayed vaginal examinations, delayed urine catheterizations, missed medications, and stressful and unpleasant perceived treatments by the staff. Several women wished for increased staffing in the labor ward due to the stressful work environment.

Even though the work environment was perceived as stressful, most women expressed empathy and an understanding by the staff. Although they had a noticeably strained work environment, some women felt satisfied and safe during birth, and the staff was described as calm, safe, and professional.

On the other hand, during the postnatal period, the work situation was described as stressful and insufficient. Some women felt forgotten in their rooms and mentioned that the help they wanted did not appear.

### Desiring the continuity of carers and a functioning team

During the active phase of labor, several women described the need for staff continuity. Women who experienced some staff changes experienced insecurity. When the birth passed from the first to the second stage of labor, several women described changes in staff resulting in emotional strain and disruption in the birthing process. When the midwife remained despite shift changes, the women expressed gratitude, and the birth experience benefitted from the midwife’s action:

*‘When her shift was over, another midwife took over, but “mine” stayed until my daughter was born, and I appreciated that!’*

However, shift changes were not always described as negative. In the cases where the woman did not feel well treated by the midwife, a change in shifts meant an opportunity for improvement. A well-functioning staff team provided a positive experience and security. If the team’s cooperation did not work, most of the women felt insecure. Furthermore, the birth experience could be negatively affected by a pronounced hierarchy between the staff and those in bad moods. Some women also reported that the reporting between staff members when changing shifts could be improved.

### Strong emphasis on family-centered care

Most women wanted their partners to be more involved in childbirth. Some women described appreciation when the labor staff also encouraged and supported their partners during birth:

*‘The staff were understanding and very positive and supportive. They told the father that he did a good job and encouraged him.’*

Women had requests for increased sympathy from their partners, but practical requests such as sleeping accommodation and food for the partner were considered necessary. During the postnatal period, several women described the need for support from their partner and increased the partner’s involvement. The most prominent wish was to give the partner the opportunity to stay with the woman and newborn child in the postnatal ward. Women described longing to be together with their newborns and partners to share the first time together. To enable this, single rooms were desirable:

*‘After a long birth, to have access to sleep and not have to share a room with someone unknown so the father can stay overnight. The mother never gets a chance to sleep but gets tense and exhausted.’*

## DISCUSSION

This study aimed to describe women’s preferences and experiences concerning the support and treatment they received and their perceptions of the quality of care during all phases of labor and the postnatal period. The analysis yielded ten sub-categories that captured various aspects of the childbirth experience including the ability to influence care, the receipt of empathic and individualized support, and active participation in one’s childbirth. The study also underscored the significance of high-quality care and the fulfilment of practical wishes. Organizational factors, such as stressful work situations and continuity of care, were identified as crucial elements. Additionally, the study highlighted the importance of family-centered care.

Three of the four dimensions of the QPP were covered by free-text comments and confirm the actuality of these dimensions. The identity-oriented approach, physical-technical conditions, and sociocultural atmosphere were covered, but the medical-technical competence domain was not. The categories of being able to influence care, receiving empathic and individualized support, and being involved as active participants in one’s own childbirth were key to the identity-oriented dimension. The importance of high-quality care, having practical wishes fulfilled, organizational issues like stressful work situations, and continuity of care were parts of the physical-technical dimension, and the importance of family centered care was sorted under the sociocultural dimension.

These findings align with previous research showing that the relationship with midwives is pivotal to the woman’s laboring experience^22^, and is also essential for a positive labor experience^23^. Our study also demonstrated that the way intrapartum care is organized can affect women’s experiences. Women prefer high-quality care, regardless of admittance to the hospital, during all phases and stages of childbirth^11,24,25^. As stated in previous research, the high workload in the labor wards was evident for the participants and affected them negatively^26,27^. The timing of arrival at the hospital, as guided by individual needs, has also been established in previous research^28-30^ and the importance of an individualized approach was confirmed. Some women need to be admitted in early labor, while others cope better at home or by staying at a nearby patient hotel. The women’s own choice guides the core approach. The need for better postnatal support was obvious in our findings. Traditionally, labor is divided into three stages, but a fourth stage has been suggested. This stage includes the first hours after birth, a period that could benefit from more attention from caregivers. Giving those hours the status of a stage in labor may contribute to a higher priority in healthcare.

The findings also showed that women were affected by the birthing environment and asked for alternatives to regular technocratic birth rooms to focus more on the physiological aspects of the birth. Findings from a Swedish project in which a birthing room was designed to be adaptable to personal wishes and needs during birth, showed that the physical features of the birthing room were important, and strengthened the view that the birthing women and care providers were those forming the birth environment and who have the major responsibility for creating safe birth outcomes^31,32^.

The women in our study wished for continuous empathic support during birth, but also asked for more support both in early labor and postnatally. Continuous support during childbirth may improve outcomes for both women and children, including more spontaneous vaginal births, shorter durations of labor, decreased use of analgesia, and more positive childbirth experiences^33^. As highlighted in the meta-synthesis by Bohren et al.^33^, women who experience continuous support especially value receiving informational support, advocacy, practical support, and emotional support, as also mentioned by the women in the free-text comments. The importance of family centered care was another finding from the present study. Women appreciated if their partners were acknowledged, and the partner’s needs were met. These findings are stated in a meta-synthesis^34^ and family centered care was established as an important part of high-quality care in a review^35^ and further emphasized in a document from WHO^36^.

None of the categories was included in the medical-technical competence domain in the present study. This can be discussed about research showing that birthing women have high trust in receiving professional medical care^18,37^. Women value a birthing environment that lets them use their inherent physical and psychosocial capacities and want to give birth to a healthy baby in a safe environment that provides reassurance and technical competency^38^. In a high-quality setting like Sweden, this is taken for granted, but ongoing discussions about informed consent can add nuance to the situation and improve the women’s security and satisfaction. ‘Birth Rights Sweden’ discusses this in a newly published report and highlights examples of disrespectful care during pregnancy, labor, and the postnatal period^39^.

### Strengths and limitations

The results of this study are based on free-text comments, and the method does not offer in-depth responses as in interviews. However, by mixing an inductive analysis and deductive approach, a more reliable interpretation is possible, and the result is additionally validated using an existing framework such as QPP. Furthermore, the order and context of the questions could have influenced the women’s comments, but when asked to state the best ways to improve the approach, both positive and negative comments were possible. The generalizability is improved by the large sample of women with negative and positive experiences. However, more primiparous women responded to the free-text comments, which could be seen as a limitation. Another limitation is that the sample was based on women giving birth in only one labor setting. However, a larger group of women were invited by including a one-year cohort.

## CONCLUSIONS

Our findings show that women view the quality of care in connection with childbirth as seamless and that they lack support mainly during the latent phase of labor as well as during the early postnatal period. Three of the dimensions in quality from the patient perspective were covered in the categories, which confirms the framework’s actuality. The quality of care from the patient’s perspective is very important when the improvement of quality of care is needed. Support and care are mostly valued, and individualized care was emphasized. Organizational structures influenced care and were more pronounced for birthing women. By including women’s involvement in their care, a more person-centered approach is possible and strengthened. Women consider all care in connection with childbirth as important. More research is needed about all phases during labor and birth, including the postnatal phase, to improve the total childbirth experience.

## Data Availability

The data supporting this research are available from the authors on reasonable request.
